# Development, characterization, and application of a 2‐Compartment system to investigate the impact of pH inhomogeneities in large‐scale CHO‐based processes

**DOI:** 10.1002/elsc.202000009

**Published:** 2020-05-28

**Authors:** Katrin Paul, Katharina Böttinger, Bernd M. Mitic, Georg Scherfler, Christoph Posch, Dirk Behrens, Christian G. Huber, Christoph Herwig

**Affiliations:** ^1^ Institute of Chemical Environmental and Bioscience Engineering TU Wien Vienna Austria; ^2^ Christian Doppler Laboratory for Mechanistic and Physiological Methods for Improved Bioprocesses TU Wien Vienna Austria; ^3^ Department of Biosciences Bioanalytical Research Labs University of Salzburg Salzburg Austria; ^4^ Christian Doppler Laboratory for Innovative Tools for Biosimilar Characterization University of Salzburg Salzburg Austria; ^5^ Sandoz GmbH Langkampfen Austria

**Keywords:** 2‐Compartment system, CHO, inhomogeneities, large‐scale, pH excursions, scale‐down

## Abstract

Large‐scale bioreactors for the production of monoclonal antibodies reach volumes of up to 25 000 L. With increasing bioreactor size, mixing is however affected negatively, resulting in the formation of gradients throughout the reactor. These gradients can adversely affect process performance at large scale. Since mammalian cells are sensitive to changes in pH, this study investigated the effects of pH gradients on process performance. A 2‐Compartment System was established for this purpose to expose only a fraction of the cell population to pH excursions and thereby mimicking a large‐scale bioreactor. Cells were exposed to repeated pH amplitudes of 0.4 units (pH 7.3), which resulted in decreased viable cell counts, as well as the inhibition of the lactate metabolic shift. These effects were furthermore accompanied by increased absolute lactate levels. Continuous assessment of molecular attributes of the expressed target protein revealed that subunit assembly or *N*‐glycosylation patterns were only slightly influenced by the pH excursions. The exposure of more cells to the same pH amplitudes further impaired process performance, indicating this is an important factor, which influences the impact of pH inhomogeneity. This knowledge can aid in the design of pH control strategies to minimize the effects of pH inhomogeneity in large‐scale bioreactors.

**Abbreviations**
1/2‐CS: 1/2 Compartment system
CHO: Chinese Hamster ovary
NaOH: sodium hydroxide
VCC: viable cell concentration

## 1 INTRODUCTION

In 2013, 10 tons of monoclonal antibodies (mAbs) were produced to meet the therapeutic demand [1]. Particularly mAbs like adalimumab (Humira), of which extensive quantities are needed [2], can be produced more economically in large‐scale bioreactors. These large‐scale reactors can reach volumes of up to 25 000 L and with increasing volume, the mixing time of the reactor also increases [3]. This in turn can lead to the formation of gradients in pH, substrate, and dissolved gasses [4]. These differences between the reactor scales can negatively influence process performance and therefore product quality attributes such as *N*‐linked glycosylation. Decreased cell counts were reported when scaling up a process from 3 to 25 000 L [5], while increased maximal lactate levels were observed during the scale up from 200 to 15 000 L [6] and when scaling up from 2 to 600 L [7]. Particularly changes in lactate metabolism have been implied to be a process predictor in the early stages of a cultivation [8], while lactate consumption was identified as the main factor to predict the final process outcome in an analysis of almost 250 cultivations [9]. This shows that changes in lactate metabolism can correlate to impaired process performance and therefore be an indicator that process scale‐up was not optimal. Hypoxia [10], as well as a combination of bad mixing and CO_2_ build up [11] have been identified as contributors to poor process performance at large scale. Particularly, increased CO_2_ concentrations have been implicated to be correlated with increased lactate levels [12, 13]. Since a higher pH is also associated with higher lactate levels [14, 15], the intermittent exposure to an increased pH at the point where base is added from the top of a large‐scale reactor can also be potentially responsible for increased lactate levels. The characterization of occurring pH inhomogeneities of an 8000 L bioreactor for cell culture revealed that amplitudes of up to 0.4 pH units can occur, when the reactor is aerated, while approximately 5% of the cell population is exposed to the increased pH [16, 17]. Furthermore, it has been determined that cells are exposed to inhomogeneous areas for the circulation time of the reactor [18]. Studies to determine the influence, which pH inhomogeneities have on process performance have been conducted in regular bioreactors [19, 20], as well as 2‐Compartment Systems (2‐CS) [21, 22, 23]. The challenge in studying pH excursions in regular bioreactors lies in the fact, that each pH corrective action increases media osmolality. Since osmolalities above 400 mOsmol kg^−1^ impair cell growth [24] and can influence product quality [25, 26], the separation of effects caused by the addition of base and those caused by an increased osmolality is difficult. Furthermore, interactive effects were observed, when Chinese Hamster ovary (CHO) cells are exposed to multiple stresses simultaneously, which makes a separation of the influences of different stress parameters difficult [27]. While different types of multiple compartment systems are well established for microbial cells [28, 29, 30, 31], systems for mammalian cells are rare and usually struggle with impaired process performance even when no inhomogeneities are introduced in the system [32].

**PRACTICAL APPLICATION**

Scale up of bioprocesses can still present a challenge and small‐scale process performance cannot always be reproduced at large scale. One possible factor can be the exposure of the cells to an increased pH in the zone, where base is added to the large‐scale reactor. This can result in decreased viable cell counts and the absence of the lactate metabolic shift. To avoid these pitfalls during scale up, the pH correction strategy should be well designed. Either a continuous addition of small amounts of base, a large pH dead band or the control of the pH with sparged gases only, are all viable options.

However, 2‐CS offers the benefit of exposing only a defined proportion of the cell population to inhomogeneity and represent a large‐scale bioreactor more realistically.

The goal of this work is the development of a 2‐CS, which is able to mimic a large‐scale bioreactor at lab scale. Therefore, the 2‐CS needs to be capable of reproducing the circulation time of a large‐scale bioreactor, while exhibiting similar process performance as a regular bioreactor, when no inhomogeneity is introduced. This represents a novelty to previously established 2‐CS for pH‐excursion studies, which could not demonstrate comparability between their 1‐ and 2‐CS [21, 22]. With the established system, the influence of pH amplitudes up to a pH, which still supports growth, is investigated. This is a further novelty, since previous work focused on the exposure of cells to pH amplitudes of a pH of 8.0 and 9.0 [21, 23]. Based on the experimentally characterized mixing time of a large‐scale bioreactor with a volume of more than 10 000 L, the circulation time was estimated as a worst‐case scenario to be one quarter of the mixing time [33]. The inhomogeneous zone is approximated to be 7.5% of the reactor volume, since the characterization of an 8 m^3^ reactor showed a zone of 5%. Amplitudes up to 0.4 pH units were introduced in the system [16, 17], and their impact on process performance, as well as the influence of the residence time of the cells in the inhomogeneous zone was investigated.

## 2 MATERIALS AND METHODS

### 2.1 Cell line, preculture, 2‐Compartment System setup and cultivation conditions

A CHO suspension cell line, producing mAbs of the IgG type was cultivated in chemically defined medium. Preculture of the cells was performed in shake flasks at 10% pCO_2_, 36.5°C, and 143 rpm to expand the cells before inoculation. Bioreactors were inoculated to achieve an initial cell density of 5·10^5^ cells · mL^−1^.

The 1‐Compartment Systems (1‐CS) are unmodified 3 L bioreactors (Labfors, Infors, Bottmingen, Switzerland) in which dissolved oxygen (dO_2_) (VisiFerm, Hamilton, Franklin, MA, USA) and pH (EasyFerm, Hamilton, Franklin, MA, USA) were monitored with inline probes. pCO_2_ was monitored with an offgas sensor (BlueInOne, Bluesens, Herten, Germany). dO_2_, pCO_2_, and pH were independently controlled. pH control was performed with either 0.5, 1, or 2 M sodium hydroxide (NaOH) and 3% H_3_PO_4_, once the pH reached a value of 6.90. The dO_2_ (40%) and pCO_2_ (10%) were kept constant by the addition of O_2_ and CO_2_, respectively. A constant flow rate of 30 mL min^−1^ was achieved by varying the N_2_ addition. The temperature was initially set to 36.50°C and shifted to 33°C, once a cell density of 20‐25·10^5^ cells · mL^−1^ was reached. Feed A was continually fed from day 4 and Feed B started at day 6 of the cultivation. Both feeds contain mixtures of different amino acids. Glucose was fed continually after its concentration dropped below 2 g L^−1^ to reestablish glucose levels of 2 g L^−1^. Glucose levels never dropped beneath 0.5 g L^−1^. To assess the impact of the peristaltic and centrifugal pump, tubing was used to create a bypass and circulate the cells. Duplicate fed‐batch cultivations were performed to assess the impact of the peristaltic pump (Ecoline VC‐280, Ismatec, Wertheim, Germany), while a batch cultivation was performed to assess the contact‐free centrifugal pump (BPS‐200, Levitronix, Switzerland). Silicone and Pharmed tubing were used for the recirculation with the peristaltic pump at flow rates of 65 mL min^−1^ and varying flow rates starting at 52 and increasing from 78 (day 6) to 104 (day 7) and 130 mL min^−1^ (day 8). The centrifugal pump was evaluated at a flow rate of 310 mL min^−1^.

**FIGURE 1:**
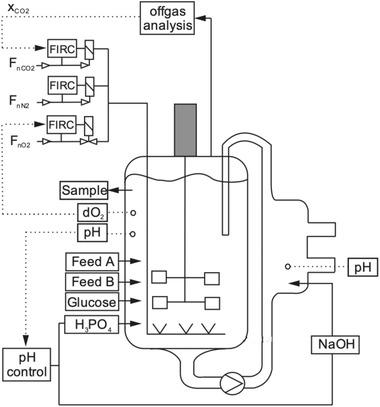
The 2‐CS setup, consisting of a bioreactor vessel with added outlet at the bottom and a bypass for the recirculation of cells

The 2‐CS is depicted in Figure 1. It consists of a 3 L bioreactor (Labfors), to which an outlet at the bottom of the reactor vessel was added, and a bypass. The bypass consists of a Mobius SensorReady Dual RS Assembly (Merck, Darmstadt, Germany), Pharmed and silicone tubing, as well as a contact‐free centrifugal pump; the system has a working volume of 2.5 L. A clamp‐on Flowsensor (Leviflow, Levitronix, Switzerland) measures the velocity of the liquid in the bypass. Control of dO_2_ and pCO_2_ was accomplished identically to the 1‐CS. pH control was implemented in the same way as for the 1‐CS for the experiment, which investigated the influence of the bypass on process performance. In the experiments with pH amplitudes, 1 M NaOH was added in the bypass, when the pH dropped below 6.88 (setpoint 6.90 with a deadband of 0.02) in the bioreactor. To achieve the desired amplitudes, experiments were performed to correlate the proportional (P) value of the PID controller to the generated pH amplitude.

### 2.2 Characterization of the 2‐CS and bioreactor with a volume of over 10 000 L

To meet distinct residence times of the cells in the inhomogeneous zone, the system was characterized with tracer pulse experiments similar to previous reports [29]. A total of 2 mL 1 M NaOH was added through a port in the bypass, while the pH was monitored in the bypass, as well as in the main reactor. The time between the increase in pH in the bypass and the increase in the main bioreactor up to 95% of the final pH is defined as the mixing time of the system. Results are fitted exponentially to determine the appropriate flow rate to achieve a mixing time of 35 and 44 s of the system.

Characterization of the large‐scale bioreactor was performed by addition of 4 M potassium chloride to the deionized water in the reactor. Conductivity was measured at three points throughout the reactor and the mixing time was calculated based on these measurements [34]. Aeration and agitation of the bioreactor were set to the operating conditions during cultivation.

### 2.3 Analytical methods

Viable cell counts (VCC), dead cell counts (DCC), and cell size were determined with the Cedex HiRes automatic picture Analyzer (Roche, Mannheim, Germany). The metabolites glucose (Glc), lactate (Lac), and ammonia (Amm) were measured with the Cedex Bio HT Analyzer (Roche). Concentration of the mAbs was measured by HPLC (Ultimate 3000, Dionex, Sunnyvale, CA, USA) with a protein A sensor cartridge (Applied Biosystems, Bleiswijk, Netherlands).

### 2.4 Estimation of the specific metabolic and product formation rates

The specific production rates for specific lactate production/consumption rate (*q*_Lac_) and specific mAb production rate (*q*_mAb_) were calculated identically with the integral viable cell density (IVCD).

(1) $$q_{\mathrm{Glc}}=\frac{{\left[\mathrm{Glc}\right]}_{1}-\;{\left[\mathrm{Glc}\right]}_{2}+\;{\left[\mathrm{Glc}\right]}_{\mathrm{Feed}}}{\left({\left[\mathrm{IVCD}\right]}_{2}-{\left[\mathrm{IVCD}\right]}_{1}\right)}$$

(2) $$q_{\mathrm{Lac}}=\frac{{\left[\mathrm{Lac}\right]}_{2}-\;{\left[\mathrm{Lac}\right]}_{1}}{\left({\left[\mathrm{IVCD}\right]}_{2}-{\left[\mathrm{IVCD}\right]}_{1}\right)}$$

(3) $$\begin{array}{ccc} {\left[\mathrm{IVCD}\right]}_{2} & = & \frac{\left({\left[\mathrm{VCC}\right]}_{2}\cdot \;V_{2}+{\left[\mathrm{VCC}\right]}_{1}\cdot \;V_{1}\right)}{2}\\ & & \cdot \;\left(t_{2}-t_{1}\right)+{\left[\mathrm{IVCD}\right]}_{1} \end{array}$$

### 2.5 Product quality (HPLC‐MS analysis)

mAb glycosylation was determined as described in a previous report [35]. The supernatant was diluted to 50 ng µ;L^−1^ with 25% acetonitrile (ACN) + 0.05% trifluoroacetic acid (TFA). Ribonuclease A (RibA) was added to a final concentration of 0.1 mg mL^−1^. A total of 0.1 mg mL^−1^ of RibA in 25% ACN + 0.05% TFA was used as a control.

Product quality was assessed on a HPLC system (Model Ultimate 3000 from Thermo Scientific, Waltham, MA) hyphenated to a quadrupole Oribtrap mass spectrometer (Model Q‐Exactive™ from Thermo Scientific™) with an Ion Max™ source (Thermo Scientific™) with heated electrospray ionization (HESI). Mass calibration was performed using the Thermo Scientific™ Pierce™ LTQ Velos ESI Positive Ion Calibration Solution for the lower mass range and 1 mg mL^−1^ ammoniumhexafluorophosphate (AHFP) in 50% methanol (MeOH) + 50% ultrapure water + 0.1% formic acid for the higher mass range. The calibration solutions were injected using a Fusion 100 infusion pump (Chemyx), a 500 µL syringe (Thermo Scientific™) for the AHFP solution, and a 250 µL syringe. mAb variants were separated on a polymeric RP column (50 × 2.1 mm i.d, 4 µm particle size MabPac RP column from Thermo Scientific™) at 70°C. The method was setup in Thermo Scientific™ Chromeleon™ 7.2.6 Chromatography Data System software. The mobile phases consisted of 0.05% TFA in ultrapure water (A) and ACN (B). The gradient elution started with 25% B for 1 min followed by a linear increase to 35% B for 1 min, and a second linear increase to 45% B within 4 min. Mobile phase B was increased to 80% for 2 min, followed by 7 min equilibration at 25% B. The flow rate was set to 300 µL min^−1^ and the injection volume was 5 µL. UV‐detection was performed at 214 nm using a 1.4 µL flow cell. Samples were measured in technical triplicates, followed by one blank run. To assess repeatability of the method and instrument performance, the quality control sample was run in the beginning, in‐between, and at the end of the sequence. Mass spectrometric data were acquired in full‐scan mode within a range of *m/z* 1800–5500 at a resolution of 17 500 at *m/z* 200 with 10 microscans being averaged in positive polarity at an in‐source collision induced dissociation of 80.0 eV. The automatic gain control (AGC) target was set to 3e6 and the maximum injection time (IT) to 150 ms. Sheath, auxiliary, and sweep gas flow rates were set to 15, 5, and 0, respectively. Spray voltage was 4 kV and S‐lens radio frequency (RF) level was 80.0. The capillary and auxiliary gas heater temperature were set to 300 and 250°C, respectively.

## 3 RESULTS AND DISCUSSION

### 3.1 Development and characterization of the 2‐CS

The first step in the establishment of the 2‐CS was the investigation of effects, which are introduced merely by the recirculation of the cells through tubing by either a peristaltic or centrifugal pump, since adverse effects have been previously reported [21, 22].

**FIGURE 2:**
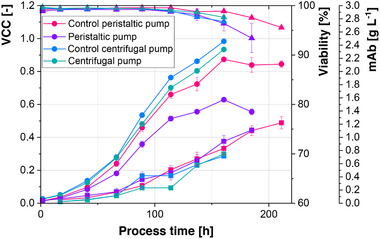
Influence of the recirculation of cells with a peristaltic and centrifugal pump. Dots represent VCC, triangles viability, and squares mAb concentration

Figure 2 shows the results of these cultivations, which revealed an approximately 27% decreased maximal VCC, when cells were recirculated with a peristaltic pump. Cell viability varied in both recirculation experiments with the peristaltic pumps, which is possibly related to the different tubing which was used. Different VCC trajectories for cells, which were circulated with a peristaltic pump, but no difference in maximal VCC was previously reported [22]. Furthermore, an earlier drop in viability, as well as a decreased specific productivity of the cells were previously observed. Since a different peristaltic pump, tubing and flow rate was used, the disparity between the observed effects can either stem from the different setup or the different cell line. However, overall process performance appears to be negatively influenced by the recirculation of the cells with a peristaltic pump, although effects vary between different setups and cell lines. This is consistent with findings correlating higher cell lysis and cell death to the use of peristaltic pumps [36, 37]. Recirculation with the centrifugal pump resulted only in a slightly lower maximal VCC (5%) at similar viability trajectories and mAb concentrations. Therefore, the centrifugal pump was chosen for the setup of the 2‐CS.

The goal of the 2‐CS was to mimic an industrial large‐scale reactor with a volume of more than 10 000 L. Its mixing time was experimentally determined to be 175 s, which is longer than for a characterized bioreactor of similar volume [38]. However, mixing times vary based on impeller configuration and operation [39]. Based on the 175 s mixing time in the reactor with a volume of more than 10 000 L, the circulation time was estimated to be 35 s (one‐fifth of the determined mixing time) and 44 s (one‐quarter of the determined mixing time) [40]. Since it has been shown that cells are exposed to inhomogeneities for a maximum of the circulation time of the reactor, the target mixing time of the 2‐CS was setup at 35 and 44s [41]. It is assumed that as soon as 95% homogeneity is achieved in the 2‐CS, the inhomogeneous zone, which is established in the bypass, disintegrated. Therefore, the cells are not exposed to inhomogeneous zone anymore, once the 2‐CS is fully mixed. This correlates to the time point in the large‐scale reactor, where the cell exits the inhomogeneous zone. Therefore, the circulation time, which represents the time throughout which a cell is exposed to the inhomogeneous zone, corresponds to the mixing time of the 2‐CS.

**FIGURE 3:**
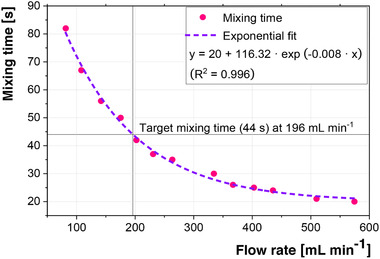
Characterization of the mixing time in the 2‐CS, based on a homogeneity of 95% in the main bioreactor

Figure 3 shows the results of the tracer pulse experiments, which were performed to characterize the mixing time of the system. The results show that the system is limited to mixing times between approximately 20 and 80s. This enables the simulation of the characterized large‐scale bioreactor with its residence time of 35 or 44 s. To ensure similar process performance between the 1‐CS and the 2‐CS, cells were recirculated in the 2‐CS, while pH corrective agents, as well as all feeds were added in the main bioreactor of the 2‐CS.

**FIGURE 4:**
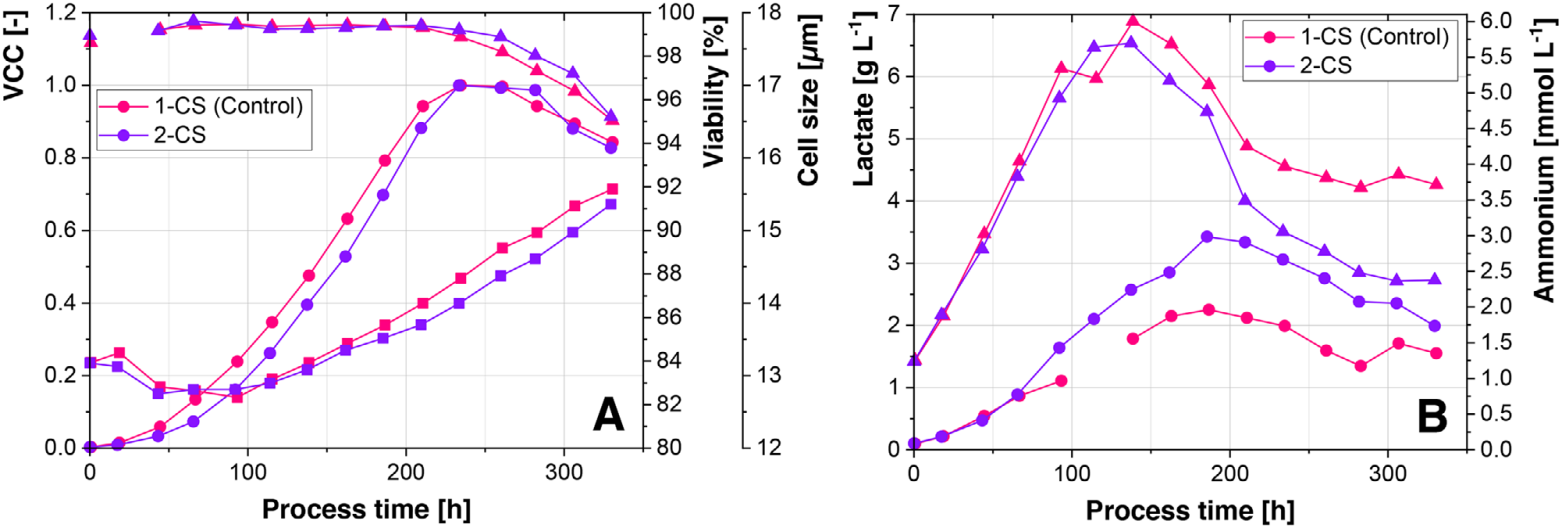
(A) Comparison of VCC (dots), viability (triangles), and cell size (squares). (B) Comparison of lactate (dots) and ammonium concentration (triangles) between the 2‐CS and the 1‐CS

In contrast to the initial Batch cultivation, where cells were only recirculated with the centrifugal pump (Figure 2), the Fed‐Batch cultivation (Figure 4A) shows no difference in maximal VCC between the 2‐CS and the 1‐CS. However, both cultivations show a slightly delayed growth of the cells in the 2‐CS. This effect is possibly related to an adaptation of the cells to the pump. Glucose, glutamine, and mAb concentrations, as well as product quality (see Appendix Figure 8) show similar trajectories throughout the process. Furthermore, the lactate metabolic shift, which is associated with metabolic efficiency and a potential predictor of process outcomes [9, 42] was observed in both cultivations and lactate consumption started at the same time in both systems. However, absolute lactate levels were 34% lower in the 1‐CS. Since increased lactate levels were also observed, when cells were only recirculated through tubing, this might be a response to the recirculation by the centrifugal pump. The effect is however possibly cell line related, since [43] observed no increased lactate levels. Lactate and ammonia uptake rates were additionally increased in the 2‐CS. Since process performance is only slightly altered between the 2‐CS and the 1‐CS, the established 2‐CS offers the benefit that any negative control does not require an additional 2‐CS, but can rather be a 1‐CS.

### 3.2 Impact of concentrated sodium hydroxide (NaOH) on process performance

To introduce amplitudes in the bypass of the 2‐CS, concentrated base is necessary. Furthermore, amplitudes are not only introduced in the bypass, but also in the main bioreactor due to the amount of base which is added to generate the amplitude. To introduce an amplitude of 1 pH unit in the bypass, for example, 2 M NaOH is necessary, while an amplitude of 0.08 units is generated in the main bioreactor. Therefore, the influence of the base strength, as well as of the pH amplitudes in the main bioreactor, on process performance was evaluated in a 1‐CS experiment.

**FIGURE 5:**
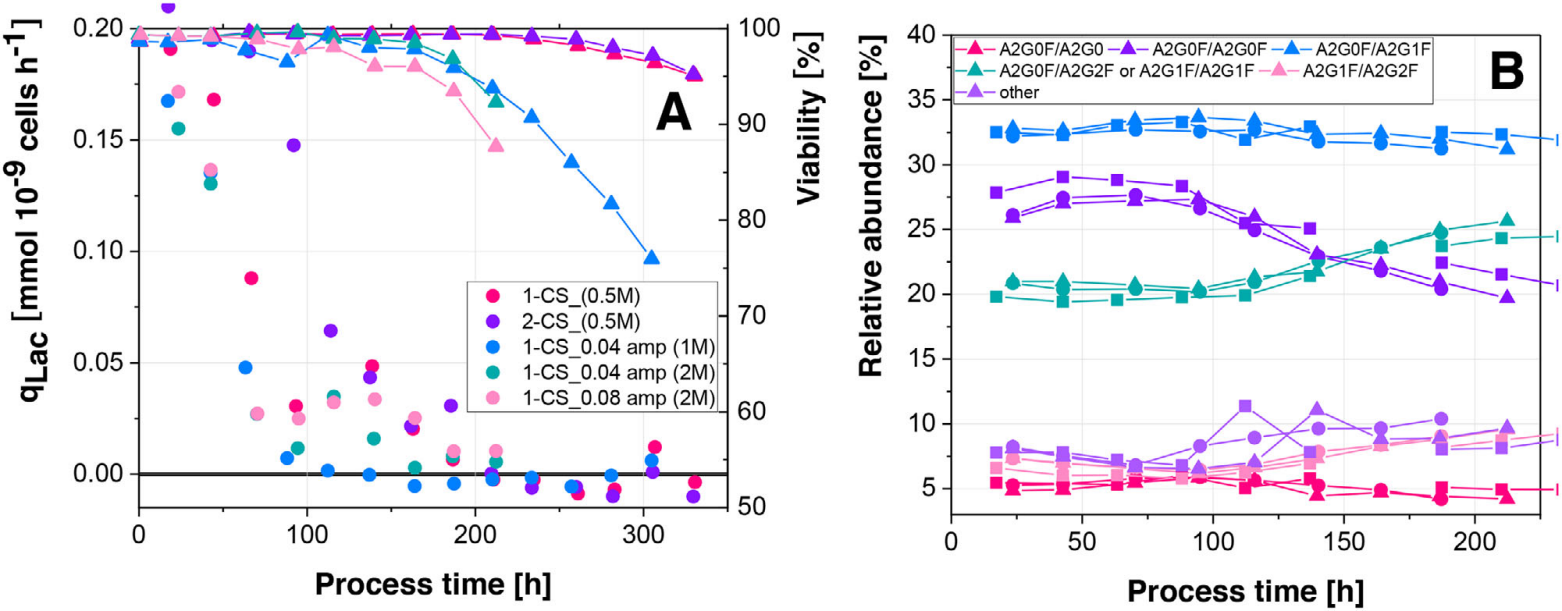
Influence of base strength and pH amplitudes on specific lactate production (circles) and viability (triangles)

In both scenarios where 2 M NaOH was used, the lactate metabolic shift was absent (Figure 5A), which indicates impaired process performance [9]. Furthermore, viability decreased earlier in the process in comparison to the control, where 0.5 M NaOH was used. When an amplitude of 0.08 units was introduced in the reactor, viability started to decrease earlier than in the scenario where an amplitude of only 0.04 units was introduced. This effect can either be related to the larger pH amplitude or to the larger amount of base which is added at one point. A total of 1 M NaOH showed no influence on the lactate metabolic shift, although the same amplitude, as for 2 M NaOH was introduced. This shows that the use of concentrated NaOH, independent of the introduced pH amplitude, altered process performance. Additionally, viability decreased earlier than in the scenario where 0.5 M NaOH was used. Figure 5B, however, shows that despite the negative impact on process performance, the relative abundances of glycoforms are highly similar in all cultivations. Since 2 M NaOH altered process performance severely, only 1 M NaOH was used for the simulation of pH inhomogeneities. These results show that the use of concentrated base can alter process performance, particularly lactate metabolism. Since scale up is often associated with increased lactate production and a diminished or absent metabolic shift [44], the use of concentrated NaOH could potentially generate 1‐CS scale‐down simulators.

### 3.3 Application of the 2‐CS

#### 3.3.1 Impact of pH excursions on process performance

To study the effect, which pH amplitudes have on process performance, 1 M NaOH was directly added into the bypass, when the pH dropped beneath the setpoint (pH of 6.88) in the bioreactor. The flow rate, as well as the pH controller were varied in both experiments to achieve similar magnitudes of the amplitudes in the bypass, while creating different residence times (44 s for 2‐CS1 and 35 s for 2‐CS2) of the cells in the inhomogeneous zone. In this way, the effects in regard to the differences in approximation of the circulation time (either one‐ fourth or one‐fifth of the mixing time) were investigated.

**FIGURE 6:**
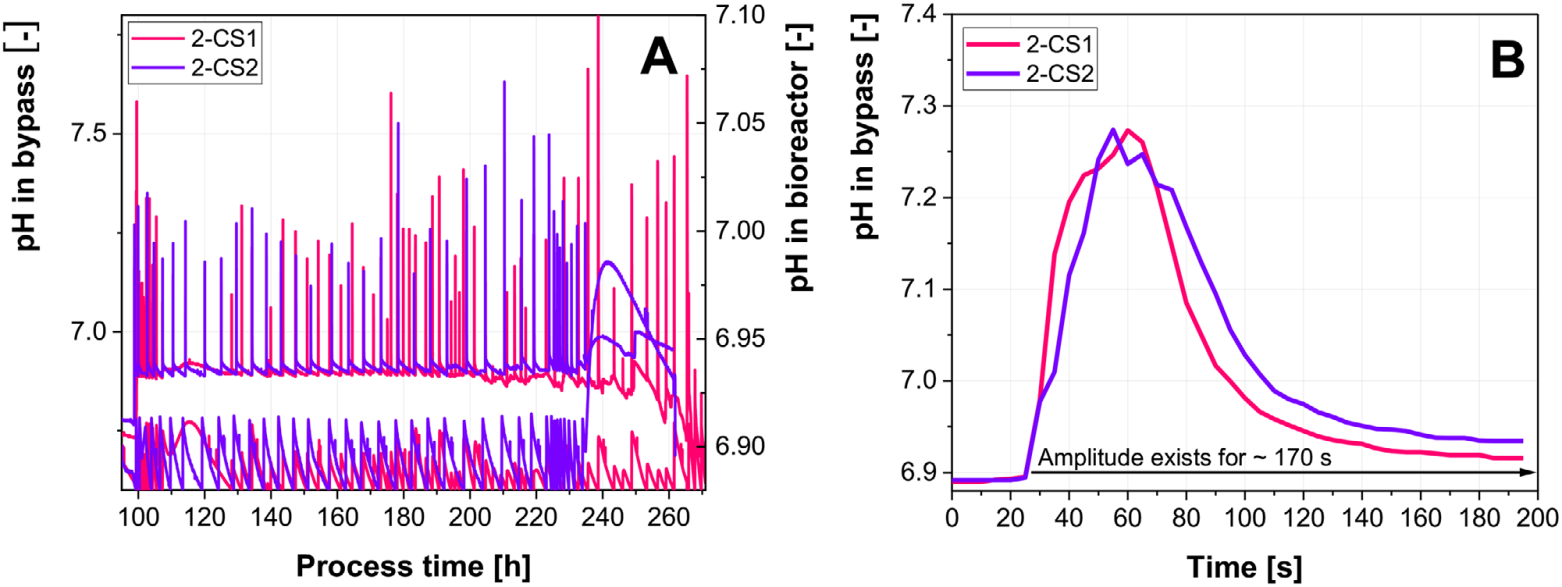
(A) pH amplitudes in the bypass (upper part of graph) and in the main bioreactor (lower part of graph) of 2‐CS cultivations run at different flow rates. (B) Single amplitudes from the bypass

Figure 6A shows the amplitudes, which were generated throughout both experiments. In the 2‐CS1, 66 amplitudes with an average magnitude of 0.36 ± 0.18 units (pH of 7.25 ± 0.18) were observed, while 2‐CS2 showed 38 amplitudes with magnitudes of 0.39 ± 0.10 units (pH of 7.28 ± 0.10). Throughout the process time, where pH amplitudes were introduced in 2‐CS2, 53 pH amplitudes occurred in 2‐CS1. The double amount of base was added with each pH correction in 2‐CS‐2 to achieve similar pH excursions in the bypass. This can be seen at the bottom of the graphs in Figure 6A, where the pH excursion in the bioreactor is twice as high for 2‐CS2 in comparison to 2‐CS1. Therefore, less amplitudes were expected in 2‐CS2. Despite the differences in both 2‐CSs, the frequency of the introduced amplitudes is similar in both experiments throughout the majority of the process. The average amplitudes are also similar, with a greater standard deviation in 2‐CS1. Figure 6B depicts a pH amplitude of the average magnitude in both systems. Despite the different flow rates, the amplitudes are similar and both amplitudes exist for approximately 170 s. This implies that in the 2‐CS2 more cells are exposed to the increased pH due to the higher flow rate, while their residence time in the inhomogeneous zone is decreased, representing the lower approximation of the circulation time. Overall, the duration of the amplitudes matched the determined mixing time of the large‐scale reactor. Therefore, the residence time of the cells in the inhomogeneous zone, as well as the time frame throughout which an inhomogeneous zone is expected can be replicated to match the characteristics of the characterized industrial large‐scale reactor in the established 2‐CS.

**FIGURE 7:**
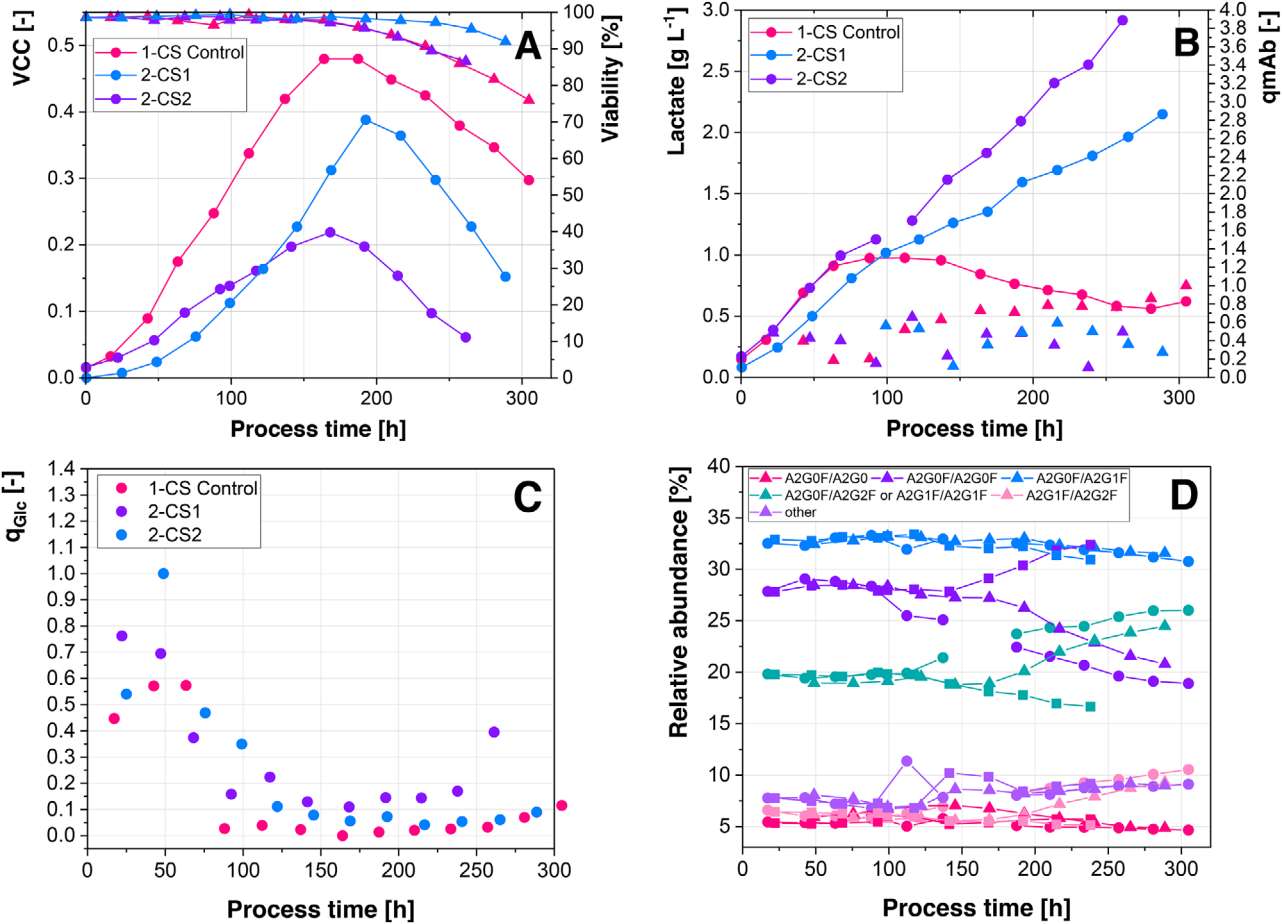
(A) VCC (dots) and viability (triangles). (B) Absolute lactate concentration (dots) and specific antibody production rate (triangles). (C) Specific glucose uptake rates of the control and the 2‐CS where pH amplitudes were introduced

Process performance varied between the different 2‐CSs. In both cases, the maximal viable cell density was decreased in comparison to the control (Figure 7). While 2‐CS1 showed a decreased maximal VCC of 18%, 2‐CS2 showed a decrease of 52%. The pH dropped to 6.8 at 100 h, at which point the pH probe was recalibrated in 2‐CS2, which likely marginally decreased the maximal VCC. Despite the considerable difference in maximal cell counts between the control and 2‐CS2, viability trajectories are similar, while the 2‐CS1 showed a slower decrease in viability and overall higher viabilities. However, the VCC trajectory of the 2‐CS1 showed a steeper decrease then the control and 2‐CS2, indicating that cells are undergoing rapid cell lysis, rather than initially becoming necrotic. Cells in both 2‐CS showed no metabolic shift to lactate consumption. The absence of the lactate metabolic shift shows that the introduction of pH amplitudes results in an altered and less efficient cell metabolism. This is also reflected in the specific glucose uptake rates (Figure 7C), which drop sharply in the control, after the temperature is lowered, while only slowly decreasing in both 2‐CS. 2‐CS2 shows higher specific glucose uptake rates than 2‐CS1, which correlates with the observed lactate levels, which are twice as high in 2‐CS1 and triple as high in 2‐CS2 in comparison to the control. Under the consideration that merely the recirculation of the cells in the 2‐CS increased lactate levels, 2‐CS1 showed a 1.5‐fold increase and 2‐CS2 showed a twofold increase in maximal absolute lactate levels. Although maximal lactate levels in 2‐CS2 were almost 1 g L^−1^ higher than in 2‐CS1, total base addition was similar throughout the process, while 53% less base was added to the control reactor. The difference in base addition is similar to that of the negative control and the 2‐CS with only recirculated cells. This shows that the absence of the lactate metabolic shift, as well as the increased lactate levels are not related to increased base addition. Furthermore, osmolality is similar between the control (305 ± 12 mOsmol kg^−1^), 2‐CS1 (321 ± 19 mOsmol kg^−1^), and 2‐CS2 (325 ± 27 mOsmol kg^−1^), showing that increased osmolality is also not the culprit for the increased lactate levels. Bolus, rather than continuous base addition has been shown to result in increased lactate levels [19], which is in alignment with the observed results. It was furthermore shown that with increasing amounts of one bolus shot, while achieving similar pH amplitudes, the maximal VCC decreased. These results are similar to the observation that 2‐CS2, where double the amount of base was added with each pH correction, showed higher lactate levels and a decreased VCC. Therefore, the increased amount of base, which is added or the increased number of cells, which is exposed to the higher pH is the determining factors of the extent to which process performance is altered. Furthermore, cells in 2‐CS2 were exposed to fewer pH amplitudes than in 2‐CS1, eliminating the number of amplitudes as a cause for worse process performance. However, previous reports showed an increased decline of the VCC with an increasing number of pH amplitudes [23]. Since aforementioned study investigated the number of pH amplitudes in a range between 10 and 100 pH amplitudes per cultivation, it is likely that the influence of the frequency of amplitudes between the 2‐CSs is too minor in comparison to the influence of the increased number of exposed cells in 2‐CS2. The obtained results highlight the benefit of the 2‐CS in comparison to a 1‐CS, where the frequency of and the extent of the amplitudes are correlated to the amount of base added, while the 2‐CS offers the benefit of decoupling these effects.

#### 3.3.2 Impact of pH excursions on product quality

The changes in process performance impacted the glycovariants of the mAb in both 2‐CSs (Figure 7D). Glycovariants are similar in all conditions until 100 h, when base addition and therefore pH excursions started to occur. Trajectories for both 2‐CSs are similar for approximately 48 h after the base addition started. At this point, 2‐CS1 starts to follow the trajectory of the control and the galactosylation (A2G1F/A2G2F and A2G0F/A2G2F or A2G1F/A2G1F) increases in the control and 1‐CS1. The highest galactosylated mAb (A2G1F/A2G2F) remained at approximately 5% throughout the process in 2‐CS2, while the glycoforms A2G0F/A2G2F or A2G1F/A2G1F decreased from 20 to 17%. Although the overall trajectories of the galactosylated variants match between the control and 2‐CS1 after 175 h, there are differences of approximately 2% between the A2G0F/A2G2F or A2G1F/A2G1F and the galactose‐free variants in the final product. In previous studies, where different cell lines were used, contradictory observations were reported, associating increased levels of galactosylation either with decreased [45] or increased pH [19]. This indicates that these responses are cell‐line specific. It has however been consistently shown that a reduced specific mAb production rate correlates to increased galactosylation levels. This has been hypothesized to be connected to a longer residence time of the mAbs in the Golgi apparatus [15, 19, 45, 46]. The specific productivity of the cells is decreased in both 2‐CS in comparison to the control (Figure 7B), which is in good agreement with another 2‐CS study [20]. Galactosylation levels are however decreased in both 2‐CS, with almost 12% more galactose‐free variants (A2G0F/A2G0F) in 2‐CS2 and 2% in 2‐CS1. Since aforementioned studies exposed cells to a certain pH for prolonged periods, it appears that the transient exposure to an increased pH affects cells differently. While the cells possess the ability to adapt to different pH setpoints, it is likely that they have only limited abilities to adapt to the transient exposure of an increased pH. Since the A2G0F/A2G2F or A2G1F/A2G1F variants in 2‐CS1 increase after an initial decrease, when base addition was started, a certain degree of adaptability is however apparent. However, an adaptation of the cells was not observed in 2‐CS2, where aglycosylated glycovariants are steadily increasing throughout the process. Therefore, either the increased amounts of added base, or the increased number of exposed cells to the higher pH, negatively influenced the adaptability of the entire cell population. Overall, molecular changes in the target mAb were mostly observed at the level of galactosylation.

## 4 CONCLUDING REMARKS

The established 2‐CS was designed to mimic a characterized industrial large‐scale bioreactor with a volume of more than 10 000 L and an experimentally determined mixing time of 175 s. Comparable process performance was observed between the 2‐CS and an unmodified lab‐scale bioreactor, which has not been the case for other described systems. This offers the benefit of using the unmodified lab‐scale bioreactor as a negative control, as well as a better separation of the effects caused by the introduction of inhomogeneity. The 2‐CS was used to investigate how zones with an increased pH, which are the consequence of base addition from the top of a large‐scale bioreactor, affect process performance. In this study amplitudes of a magnitude of 0.4 pH units were investigated. Additionally, the effects of two different residence times of the cells in the inhomogeneous zone was assessed, since the circulation of a bioreactor can be estimated as either one‐fourth or one‐fifth of the mixing time of the reactor. The results show that the introduction of pH amplitudes eliminated the metabolic shift from lactate production to lactate consumption, independent of the residence time of the cells in the inhomogeneous zone. However, absolute lactate levels were further increased, when more base was added during pH correction, exposing more cells to the inhomogeneous zone, despite the lower exposure time. The VCC was decreased in both experiments, but to a greater extent, when more cells were exposed to the pH excursions. Galactose‐free types were predominantly observed in this scenario as well. Therefore, the amount of base, which is added with each pH correction or the increased number of exposed cells is the determining factor to which extent process performance and product quality are influenced by pH inhomogeneity. This knowledge enables the design of pH control strategies to minimize the impact of pH inhomogeneity. One possibility would be the continuous addition of base based on process characterization studies to add minimal amounts of base with each addition. Furthermore, a wide dead band on pH control has the benefit to decrease or even eliminate base addition. This can also be achieved by controlling the pH by varying the composition of the sparged gases, rather than by base addition. Future work still needs to determine, whether the observed effects are cell‐line specific, or if a similar response can be observed for other CHO cell lines. Furthermore, the threshold at which process performance is not altered by base addition should be determined to assess the robustness of CHO cells to pH excursions.

## CONFLICT OF INTEREST

The authors have declared no conflict of interest.

## Supporting information

Figure 8: A Glucose (dots), glutamine (triangles) and mAb (squares) concentration for the recirculation of cells through the bypass and the 1‐CS control. B Glycovariants of the control (dots) and the 2‐CS (triangles).
