# Supplementary figures and images for: Development, characterization, and application of a 2‐Compartment system to investigate the impact of pH inhomogeneities in large‐scale CHO‐based processes
Source: Eng Life Sci. 2020 May 28;20(8):368–78. doi: 10.1002/elsc.202000009 (PMC7401239; doi:10.1002/elsc.202000009)

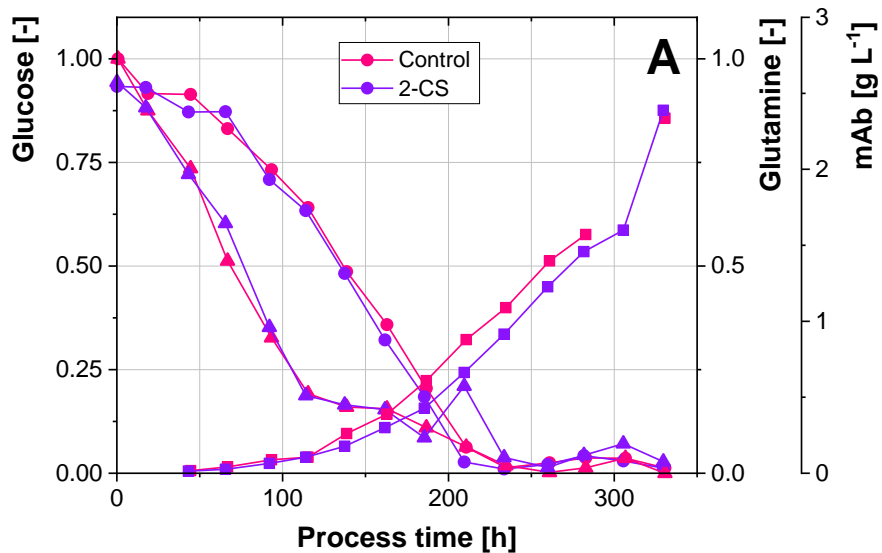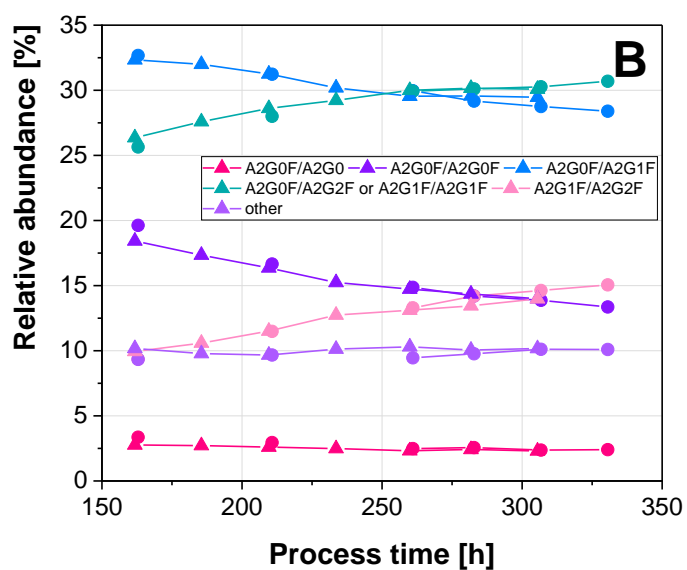

Supplement: Supplementary file 1 — Figure 8: A Glucose (dots), glutamine (triangles) and mAb (squares) concentration for the recirculation of cells through the bypass and the 1‐CS control. B Glycovariants of the control (dots) and the 2‐CS (triangles). [file ELSC-20-368-s001.pdf]
